# The Combination of TRAIL and Isoflavones Enhances Apoptosis in Cancer Cells

**DOI:** 10.3390/molecules15032000

**Published:** 2010-03-22

**Authors:** Joanna Bronikowska, Ewelina Szliszka, Zenon P. Czuba, Dariusz Zwolinski, Dariusz Szmydki, Wojciech Krol

**Affiliations:** Chair and Department of Microbiology and Immunology, Medical University of Silesia in Katowice, Jordana 19, 41-808 Zabrze, Poland; E-Mails: jbronikowska@sum.edu.pl (J.B.); eszliszka@sum.edu.pl (E.S.); zczuba@sum.edu.pl (Z.P.C.); 6132@wp.pl (D.Z.); dariusz.szmydki@op.pl (D.S.)

**Keywords:** isoflavones, TRAIL, apoptosis, chemoprevention, cancer cells

## Abstract

Isoflavones are a class of bioactive polyphenols with cancer chemopreventive properties. TRAIL (tumor necrosis factor-related apoptosis-inducing ligand) is a naturally occurring antitumor agent that selectively induces programmed death (apoptosis) in cancer cells. Polyphenols can modulate TRAIL-mediated apoptosis in cancer cells. We examined the cytotoxic and apoptotic activities of isoflavones in combination with TRAIL on HeLa cancer cells. The apoptosis was detected by fluorescence microscopy with annexin V-FITC. The cytotoxicity was evaluated by MTT and LDH assays. The tested isoflavones: genistein, biochanin-A and neobavaisoflavone enhance TRAIL-induced apoptosis in HeLa cells. Our study indicated that isoflavones augmented TRAIL-cytotoxicity against cancer cells and confirmed potential role of those polyphenols in chemoprevention.

## 1. Introduction

Phenolic and polyphenolic compounds constitute one of the most numerous groups of chemicals found in the plant kingdom. They can be divided into various classes on the basis of their molecular structure, with flavonoids being one of the main groups [1]. Epidemiological studies have described the beneficial effect of dietary flavonoids on the reduction of the risk of various cancers [2,3]. The main groups of important flavonoids contained in human diet are: anthocyanindins, flavones, flavonols, flavanols, flavanones and isoflavones [1,3].

Malignant diseases are a basic public health burden in the World and one of the main causes of death in both men and women. More than 10 million people are diagnosed of cancer every year and it is estimated that by 2020 there will be some 16 million new cases per year. Chemoprevention, in which natural or synthetic agents are used to prevent malignant diseases, is one of the most promising approaches in cancer research [4,5]. 

Many phytochemical compounds have been shown to be biologically active and protect against cancer [6,7,8,9,10,11]. The human diet contains a complex mixture of plant polyphenols. Isoflavones possess cancer chemopreventive properties [7,10,12]. The major dietary source of these flavonoids are soybeans [12,13]. Significant correlations between an isoflavone rich soy-based diet and reduced incidence of breast and prostate cancer have been described [14,15]. Both *in vitro* and *in vivo* studies also showed that isoflavones induce apoptosis in cancer cells [16,17,18,19,20].

Tumor necrosis factor-related apoptosis-inducing ligand (TRAIL) belongs to the TNF protein superfamily. TRAIL induces apoptosis in various types of malignant tumor cells through its interaction with the death domain–containing receptors, TRAIL-R1 (DR4) and TRAIL-2 (DR5) with no effect on normal cells. Soluble or expressed on NK cells, lymphocytes T, monocytes or macrophages and neutrophils, molecules of TRAIL play a critical role in immune surveillance [21]. Although TRAIL has strong apoptosis activation properties, some cancer cells are resistant to TRAIL-mediated programmed death. The decreased expression of death receptors TRAIL-R1 and TRAIL-R2 or increased expression of antiapoptotic protein in cancer cells are involved in TRAIL-resistance [22]. We and others have shown that HeLa cancer cells are resistant to TRAIL-induced apoptosis [23,24,25]. TRAIL-resistant cancer cells can be sensitized to TRAIL-mediated cytotoxicity by anticancer agents such as dietary polyphenols [23,24,25,26,27].

In this study we demonstrate that three isoflavone derivatives: genistein, biochanin-A and neobaisoflavone enhanced TRAIL-mediated apoptosis in HeLa cancer cells. The TRAIL-induced apoptotic signaling pathway is a potential target for the polyphenols in tumor cells and the overcoming of TRAIL-resistance by isoflavones may be one of the mechanism responsible for their cancer chemopreventive properties [28,29]. 

## 2. Results and Discussion

Isoflavones have been the subject of intensive studies because they exert biological effects that may help to reduce the risk of developing certain diseases [14,15,30]. These subclass of flavonoids are known to possess estrogenic, antioxidant, immunomodulatory and anticancer activities [7,10,11,16,17,18,19,20,30,31,32,33,34,35,36,37,38,39]. Epidemiological findings described the beneficial effects of dietary isoflavones on cancer prevention. Doll and Peto indicated that an average of 35% of overall human death rate for cancer is associated with nutritional factors [4]. Isoflavones are found in legumes, however the richest dietary source of genistein and biochanin-A are soybeans [40]. Populations that regularly consume soy based foods (*i.e.,* Asian populations) have lower incidences of breast and prostate cancers [14,15]. Beside genistein and biochanin-A present mainly in soybean and soybased food, neobavaisoflavone is an isoflavone isolated from Leguminosae plant named *Psoralea corylifolia* [41].

### 2.1. Cytotoxic and apoptotic activity of isoflavones in HeLa cancer cells

**Figure 1:**
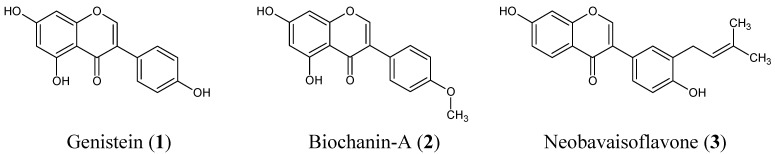
Chemical structures of the tested isoflavones.

We tested cytotoxic and apoptotic activities of three isoflavones: genistein, biochanin-A and neobavaisoflavone against HeLa cancer cells. Figure 1 presents the structures of isoflavones used in this study. Previously, experimental data showed that genistein and biochanin-A inhibit umor growth by inducing cell cycle arrest and apoptosis in cancer cells [16,17,18,19,20]. It has been also reported that the extract of *Psoralea corylifolia* exhibit anticancer properties, but there is no evidence of cytotoxic or apoptotic activity of neobavaisoflavone [42].

**Figure 2:**
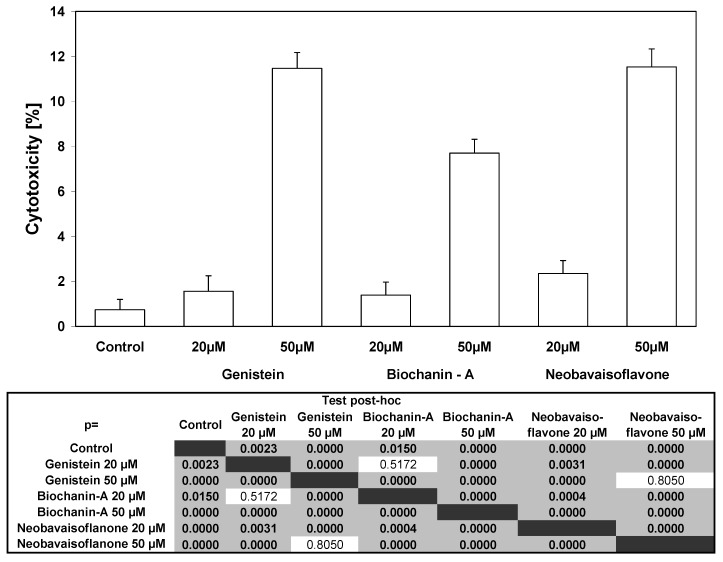
Cytotoxic activity of isoflavones in HeLa cancer cells. The cancer cells were incubated for 48 hours with three isoflavones: genistein, biochanin-A, neobavaisoflavone at the concentrations of 20 μM and 50 μM. The values represent mean ±SD of three independent experiments performed in quadruplicate (n = 12) (p < 0.05). The percentage of cell death was measured by MTT cytotoxicity assay.

We have demonstrated that the tested isoflavones at concentrations of 20–50 μM induced cytotoxicity in HeLa cells in a dose-dependent manner: 1.56 ± 0.69%–11.47 ± 0.70% cell deaths for genistein, 1.39 ± 0.58%–7.70 ± 0.62 cell deaths for biochanin-A and 2.35 ± 0.57%–11.42 ± 0.83% cell deaths for neobavaisoflavone (Figure 2). 

Our results indicated that cytotoxic effect of isoflavones against HeLa cancer cells is mediated through apoptosis. The percentage of apoptotic cells after exposure of 50 μM isoflavones were: 12.63 ± 1.06% for genistein, 8.75 ± 0.89% for biochanin-A and 12.75 ± 0.90 for neobavaisoflavone. The percentage of necrotic cells examined by lactate dehydrogenase assay and fluorescence microscopy with annexin V-FITC test was near 0%.

Isoflavones induce programmed death in tumor cells targeting several molecules associated with multiple signaling pathways [30]. Kim *et al*. showed that genistein is involved in both extrinsic and intrinsic apoptotic pathway in HeLa cancer cells. Genistein triggers apoptosis through increased truncation of Bid and Bax expression, which plays an important role in the release of cytochrome c from mitochondrium and activation caspase-3, -8 and -9 as key regulators in the observed cytotoxic effect [43]. 

### 2.2. Cytotoxic and apoptotic activity of TRAIL in HeLa cancer cells

**Figure 3:**
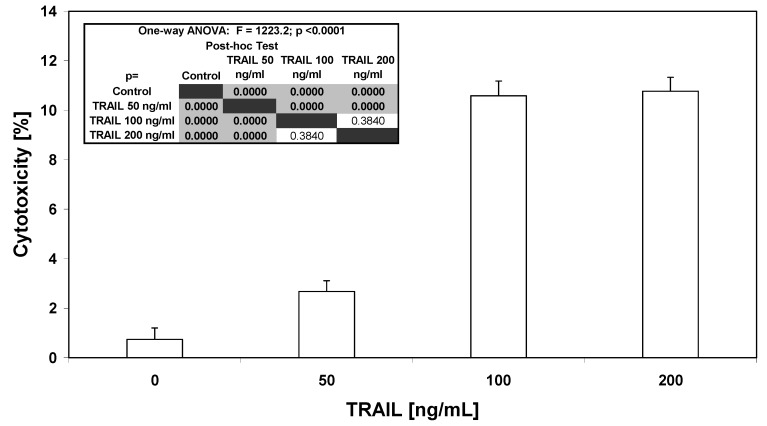
Cytotoxic activity of TRAIL in HeLa cancer cells. The cancer cells were incubated for 48 hours with TRAIL at the concentrations of 50–200 ng/mL. The values represent mean ±SD of three independent experiments performed in quadruplicate (n = 12) (p < 0.05). The percentage of cell death was measured by MTT cytotoxicity assay.

TRAIL is a potent antitumor agent that selectively induces apoptosis in cancer cells [21]. Recombinant human TRAIL used in our study is a soluble protein based on a natural endogenous ligand. We and others showed that HeLa cells are resistant to TRAIL-mediated cytotoxicity [23,24,25]. TRAIL induced cytotoxicity in HeLa cells in dose-dependent manner (2.68 ± 0.44%–10.77 ± 0.56%). Figure 3 presents the cytotoxic activity of TRAIL. TRAIL induced the cytotoxic effect in cancer cells in the apoptotic way. The necrotic cell death percentage of HeLa cells examined by lactate dehydrogenase assay and fluorescence microscopy with annexin V-FITC test was near 0%.

The apoptotic effect of TRAIL at the concentration of 100 ng/mL was 10.75 ± 1.04%. The concentration 200 ng/mL TRAIL or higher (our unpublished observations) did not significantly increase the cytotoxic and apoptotic effects on HeLa cancer cells.

### 2.3. Cytotoxic and apoptotic activity of TRAIL in combination with isoflavones in HeLa cancer cells

Several cellular mechanisms contribute to the overall cancer preventive effects of the dietary flavonoids [1,2,3,5]. Isoflavones have received considerable attention as potential chemopreventive and anticancer agents due to their diverse effects on cellular processes [16,20]. TRAIL is considered to be a tumor-selective, apoptosis-inducing cytokine and TRAIL-mediated apoptotic pathway is a potential target for chemopreventive activity of isoflavones. TRAIL-resistant cancer cells can be sensitized to TRAIL-mediated cytotoxicity by dietary flavonoids [23,24,25,26,27,28,29]. 

**Figure 4:**
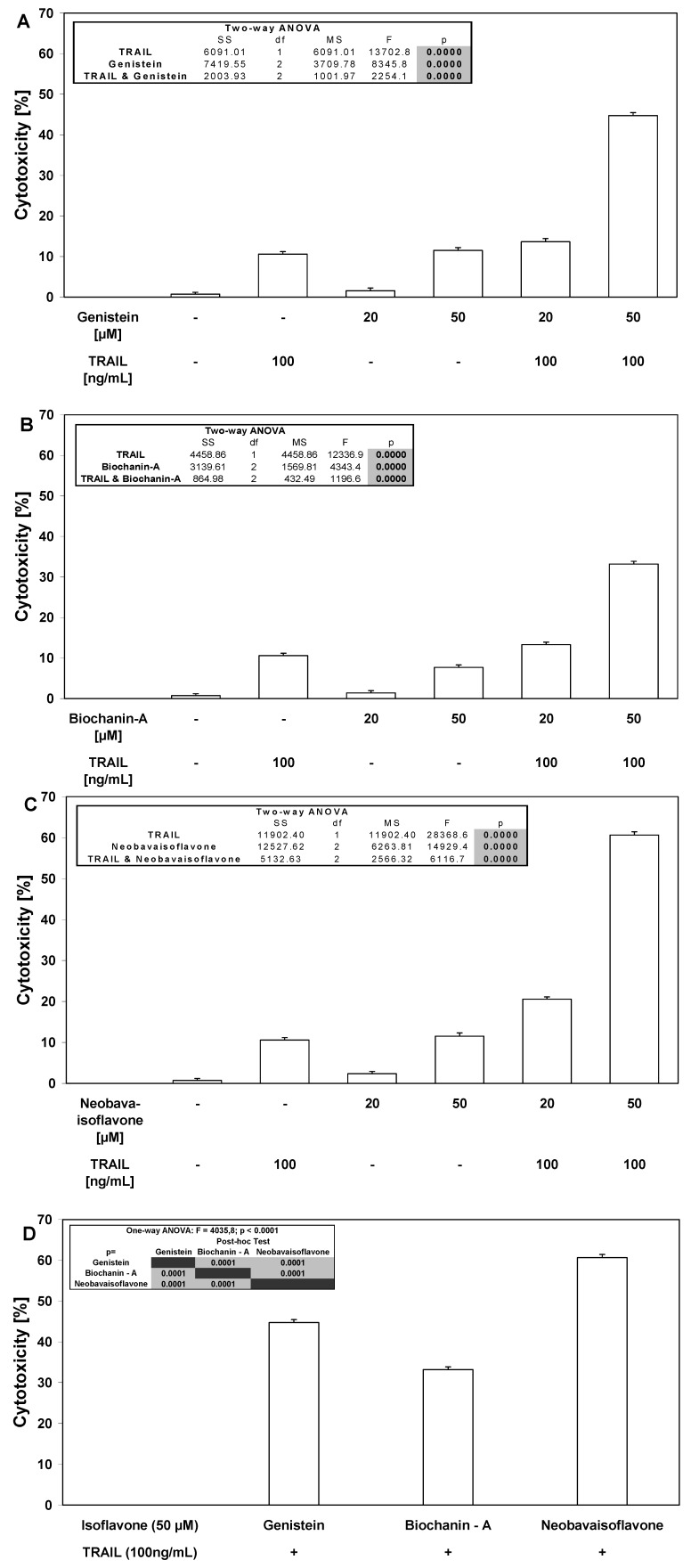
Cytotoxic activity of TRAIL in combination with isoflavones in HeLa cancer cells. The cancer cells were incubated for 48 hours with TRAIL at the concentration of 100 ng/mL and isoflavone: (**A**) genistein, (**B**) biochanin-A, (**C**) neobavaisoflavone at the at the concentrations of 20 μM and 50 μM, (**D**) comparison between isoflavones at concentration 50 μM. The percentage of cell deaths was measured by MTT cytotoxicity assay. The values represent mean ±SD of three independent experiments performed in quadruplicate (n = 12) (p < 0.05).

We investigated the cytotoxic and apoptotic activity of TRAIL in combination with isoflavones on HeLa cancer cells. The cytotoxicity of TRAIL in combination with isoflavones in HeLa cells is demonstrated in Figure 4. As shown in Figure 2 and Figure 3, little cytotoxicity was observed with isoflavones alone or TRAIL alone. HeLa cells cotreatment with TRAIL and isoflavones increased the percentage of cell deaths to 44.73 ± 0.74% for genistein, to 33.17 ± 0.70% for biochanin-A and to 60.64 ± 0.81% for neobavaisoflavone. 

Isoflavones (genistein, biochanin-A, neobavaisoflavone) in combination with TRAIL induced the cytotoxic effect in cancer cells in the apoptotic way. The necrotic cell death percentage of HeLa cells examined by lactate dehydrogenase assay and fluorescence microscopy with annexin V-FITC test was near 0%.

**Figure 5:**
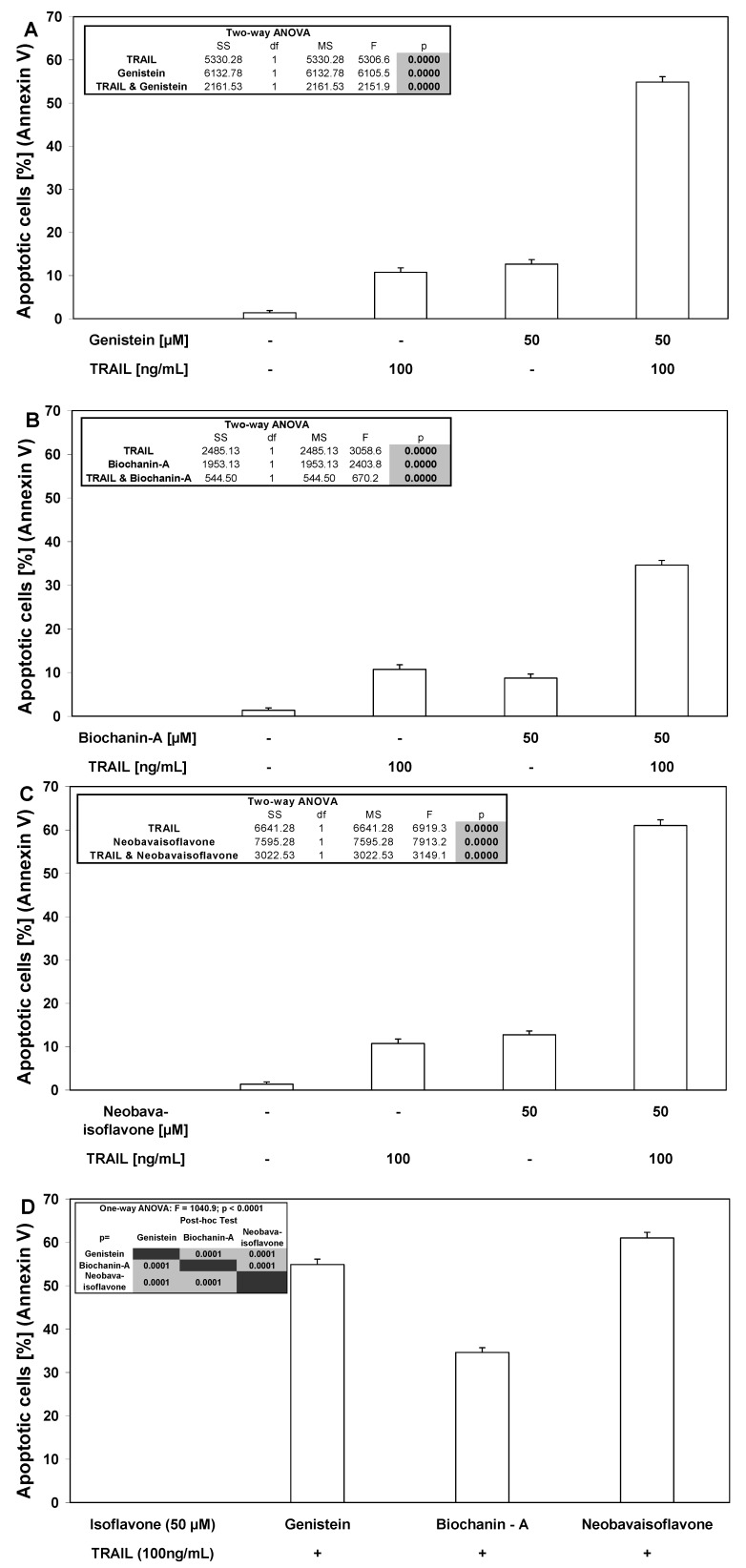
TRAIL induced apoptosis in combination with isoflavones in HeLa cancer cells. The cancer cells were incubated for 48 hours with TRAIL at the concentrations of 100 ng/mL and isoflavones: (**A**) genistein, (**B**) biochanin-A, (**C**) neobavaisoflavone at the concentration of 50 μM, (**D**) comparison between isoflavones. Detection of apoptotic cell deaths by fluorescence microscopic using annexin V-FITC, Ethidium Homodimer III and Hoechst 33342 staining. The values represent mean ±SD of three independent experiments performed in quadruplicate (n = 12) (p < 0.05).

**Figure 6:**
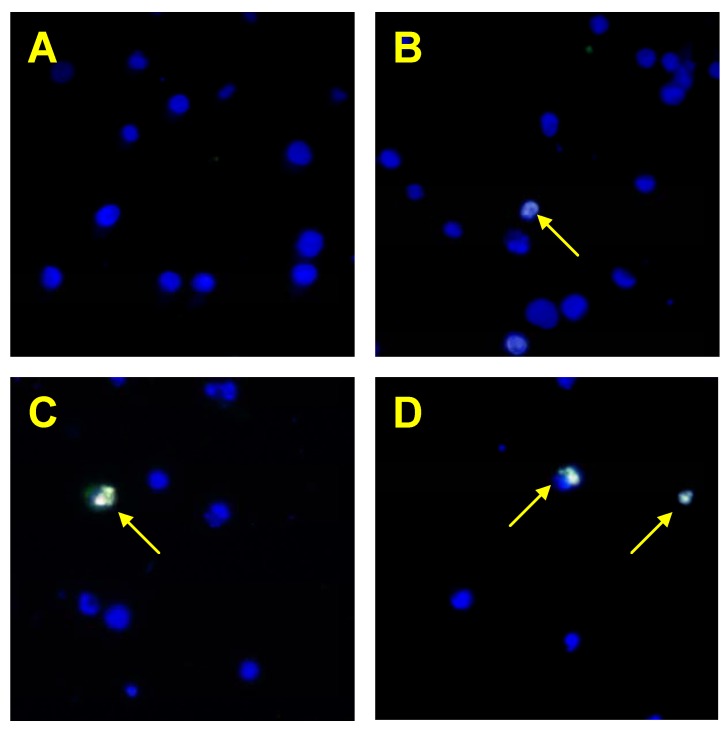
Detection of apoptotic cell deaths by fluorescence microscopic using annexin V-FITC, Ethidium Homodimer III and Hoechst 33342 staining. The healthy cells (stained with Hoechst 33342) emitted blue fluorescence and apoptotic cells (stained with Annexin V-FITC and Hoechst 33342) emitted green and blue fluorescence. (**A**) Control cells, (**B**) cells incubated with TRAIL (100 ng/mL), (**C**) cells incubated with neobavaisoflavone (50 μM), (**D**) cells incubated with neobavaisoflavone (50 μM) and TRAIL (100 ng/mL). Cells undergone apoptosis showing nuclei shrinkage, chromatin condensation and nuclei fragmentation, indicated by arrows.

Isoflavones markedly augmented TRAIL-induced apoptosis in cancer cells (Figure 5). Neobavaisoflavone exhibited the strongest apoptotic effect in combination with TRAIL (61.00 ± 1.31%) on HeLa cells (Figure 6). We have also shown that two other tested isoflavones, genistein and biochanin-A cooperate with TRAIL to induce apoptosis in cancer cells (54.88 ± 1.25% and 34.63 ± 1.06, respectively). TRAIL molecules play a significant role in immune surveillance and defense mechanisms against tumor cells [21]. Our results suggest that isoflavones can sensitize cancer cells to immune effector mechanisms such as TRAIL-mediated apoptosis. 

There are many factors contributing to the resistance to TRAIL-induced apoptosis. The decreased expression of death receptors TRAIL-R1 and TRAIL-R2 or increased expression of antiapoptotic protein in cancer cells are involved in TRAIL-resistance [22]. Therefore, further investigations will be required to explain the molecular mechanisms by which isoflavones sensitize cancer cells to TRAIL induced death. Lee *et al*. reported that tyrosine kinase inhibition by genistein in TRAIL-mediated apoptosis attenuate ERK1/2 activity and augment apoptotic effect. ERK1/2 activation via tyrosine kinase pathway plays a significant role in antiapoptotic cellular defense mechanism through the upregulation of antiapoptotic Bcl-2 protein levels in TRAIL-induced apoptosis [44].

Siegelin *et al*. described the apoptotic activity of TRAIL in combination with daidzein on glioma cells. Daidzein downregulated expression of Bcl-2 protein and overcame TRAIL-resistance in glioma cells [29]. Siegelin *et al*. investigated also apoptotic effect of cotreatment of TRAIL with genistein on glioma cells. In these authors’ opinion genistein triggers proteosomal degradation of antiapoptotic protein, cFLIP in tumor cells. Increased expression of cFLIP augmented TRAIL-mediated apoptosis in glioma cell line [45]. Nozawa *et al*. showed the enhanced TRAIL-mediated apoptosis in pancreatic cancer cells by genistein. The combined treatment of TRAIL with genistein effective inhibit the growth of pancreatic cancer *in vitro* and *in vivo*. The apoptotic effect was associated with casapase-3 activation [46]. Jin *et al*. confirmed sensitization of TRAIL-resistant gastric adenocarcinoma cells by genistein through activation of casapase-3 [28]. In other *in vitro* models, Jin *et al*. demonstrated the mechanism to overcome TRAIL-resistance after genistein exposure. Inhibition of p38-beta mitogen-activated protein kinase (MAPK) activation or promotion of Bid (Bcl-2 inhibitory protein) cleavage by genistein in TRAIL-induced apoptotic pathway restored TRAIL-sensitivity in hepatocellular carcinoma cell line [47,48]. 

The cytotoxic and apoptotic effects of biochanin-A and neobavaisoflavone in combination with TRAIL on cancer cells were examined for the first time in our study and there is no evidence of cytotoxic or apoptotic activity of TRAIL cotreatment with these isoflavones. Although many investigations have been conducted to understand the protective effects of isoflavones against cancers, the mechanism of their action are still not entilery elucidated. Enhancing TRAIL-mediated apoptosis in cancer cells by genistein, biochanin-A and neobavaisoflavone confirmed the multiple regulatory role of isoflavones in cellular signaling pathways responsible for their cancer chemopreventive properties.

In our experiment the most activity in induction of cytotoxic and apoptotic effects alone or with TRAIL was shown by neobavaisoflavone (**3**). This compound has two hydroxyl groups in the 7 and 4’ positions and a prenyl group in the 3’ position. All the tested compounds are characterized by the presence of a high reactive hydroxyl group in the 7 position in conjunction with a carbonyl group that can react with oxygen intermediates. Biochanin A (**2**) showed the lowest activity in induction of cytotoxicity and apoptosis. The structure of biochanin A differs from genistein (**1**) in the the substituent in the 4’ position. The presence of a methoxyl group in the 4’ position (biochanin A) instead of a hydroxyl group (genistein) decreased the activity of the former. The main structure of isoflavone with two hydroxyl groups (4’ and 7 positions) is similar in structure to 17β-estradiol (E2). Isoflavones are labeled as phytoestrogens that may react with estrogen receptors [49].

## 3. Experimental

### 3.1. Isoflavones

The tested isoflavones: genistein (**1**), biochanin-A (**2**), neobaisoflavone (**3**) were obtained from Alexis Biochemicals (San Diego, CA, USA) and Sigma Chemical Company (St. Louis, MO, USA). The isoflavones were dissolved in DMSO to get the final concentration.

### 3.2. TRAIL

Soluble recombinant human TRAIL (rhsTRAIL) was purchased from PeproTech Inc. (Rocky Hill, NJ, USA).

### 3.3. Cancer cell culture

The experiments were performed on a HeLa human cervical cancer cell line (DSMZ (Deutsche SammLung von Mikroorganismen und Zellkulturen) GmbH – German Collection of Microorganism and Cell Cultures, Braunschweig, Germany). The HeLa cells were grown in monolayer cultures in RPMI 1640 containing 10% fetal bovine serum (FBS) with 4 mM L-glutamine, 100 U/mL penicillin and 100 μg/mL streptomycin. The cancer cells were grown at 37 °C in atmosphere with 5% CO_2_ [23,24]. All reagents for cell culture were obtained from PAA The Cell Culture Company (Pasching, Austria).

### 3.4. Determination of apoptotic cell death by fluorescence microscopy with annexin V-FITC staining

Apoptotic cells were quantified by the fluorescence microscopy method using the Apoptotic & Necrotic & Healthy Cells Quantification Kit from Biotium, Inc. (Hayward, CA, USA) according to the manufacturer’s instruction [24]. The HeLa cells (2.5 × 10^5^/mL) were seeded 24 hours before the experiments in a 24-well plate. Various combinations of isoflavones (20–50 μM) with or without TRAIL (50–200 ng/mL) were added to the cancer cells, and 48 hours later the cells were washed with PBS and detached from cell culture wells by trypsin. Next, the HeLa cells were centrifuged to discard supernatant, washed with PBS and resuspended in Binding Buffer (100 μL/sample). To each tube were added: 5 μL of Annexin V-FITC, 5 μL of Ethidium Homodimer III and 5 μL of Hoechst 33342 solutions. The samples were incubated at room temperature for 15 minutes in the dark. After staining the cancer cells were washed with Binding Buffer and placed on a glass slide and covered with a glass coverslip. The stained cells were observed under a fluorescence inverted microscope IX51 (Olympus, Tokyo, Japan) using filter set for FITC, TRITC and DAPI. The healthy cells (stained with Hoechst 33342) emitted blue fluorescence, apoptotic cells (stained with Annexin V-FITC and Hoechst 33342) emitted green and blue fluorescence and necrotic cells (stained with Ethidium Homodimer III and Hoechst 33342) emitted red and blue fluorescence. Cancer cells stained with triple colors blue, red and green, were dead cells progressing from apoptotic cell population. The cells were counted and apoptotic cells were expressed as percentage of total cells.

### 3.5. Cytotoxixity assay

The cytotoxicity was measured by the 3-[4,5-dimethylthiazol-2-yl]-2,5 diphenyltetrazolium (MTT) assay as described [23,24,26,27,50]. The HeLa cells (5 × 10^4^ per well) were seeded 24 hours before the experiments in a 96-well plate. Various combinations of isoflavones (20–50 μM) with or without TRAIL (50–200 ng/mL) were added to the cells, and 48 hours later the medium was removed and 20 μL MTT solutions prepared at 5 mg/mL (Sigma Chemical Company) were added to each well for 4 h. The resulting crystals were dissolved in DMSO. Controls included native cells and medium alone. The spectrophotometric absorbance of each well was measured using a microplate reader (ELx 800, Bio-Tek Instruments Inc., Winooski, VT, USA) at 550 nm. The percent cytotoxicity was calculated by the formula: percent cytotoxicity (cell death) = (1- [absorbance of experimental wells/absorbance of control wells]) × 100%.

### 3.6. Lactate dehydrogenase release assay

Lactate dehydrogenase (LDH) is a stable cytosolic enzyme that is released upon membrane damage in necrotic cells. LDH activity was measured using a commercial cytotoxicity assay kit (Roche Diagnostics GmbH, Mannheim, Germany), in which LDH released in culture supernatants is measured with a coupled enzymatic assay, resulting in conversion of a tetrazolium salt into red formazan product. The HeLa cells were treated with various concentrations of isoflavones (20–50 μM) alone and in combination with TRAIL (50–200 ng/mL) for the indicated period of time. The sample solution (supernatant) was removed and LDH released from cells was measured in culture medium. The maximal release was obtained after treating control cells with 1% Triton X-100 (Sigma Chemical Company) for 10 minutes at room temperature [23,24,26,27,50]. The necrotic percentage was expressed using the formula: (sample value/maximal release) × 100%.

### 3.7. Statistical analysis

The results are expressed as means ±S.D. obtained from three separate experiments performed in quadruplicate (n = 12) for cytototoxicity or duplicate (n = 6) for apoptosis. The experimental means were compared to the means of untreated prostate cancer cells harvested parallelly and the data was polled for replicate experiments. Statistical significance was evaluated using one- and multiple-way ANOVA or Kruskal-Wallis test. P-values < 0.05 were considered significant.

## 4. Conclusions

Isoflavones exert chemopreventive and antitumor activities through regulation of different cell signal transduction pathways that are involved in the development and progression of cancer. Our study indicated that the tested isoflavones – genistein, biochanin-A and neobaisoflavone – enhance TRAIL-induced apoptosis of HeLa cancer cells.

## References

[1] Han X., Shen T., Lou H. (2007). Dietary polyphenols and their biological significance. Int. J. Mol. Sci..

[2] D’Archivio M., Santangelo C., Scazzocchio B., Vari R., Filesi C., Masella R., Giovannini C. (2008). Modulatory effects of polyphenols on apoptosis induction: Relevance for cancer prevention. Int. J. Mol. Sci..

[3] Pereira D.M., Valentão P., Pereira J.A., Andrade P.B. (2009). Phenolics: From chemistry to biology. Molecules.

[4] Doll R., Peto R. (1981). The causes of cancer: Quantitative estimates of avoidable risk of cancer today. J. Natl. Cancer Inst..

[5] Ramos S. (2007). Effects of dietary flavonoids on apoptotic pathways related to cancer chemoprevention. J. Nutr. Biochem..

[6] Gazak R., Sedmera P., Vrbacky M., Vostalova J., Drahota Z., Marhol P., Walterova D., Kren V. (2009). Molecular mechanisms of silybin and 2,3-dehydrosilybin antiradical activity-role of individual hydroxyl groups. Free Radic. Biol. Med..

[7] Perabo F.G., Low E.C., Ellinger J., Rücker A., Müller S.C., Bastian P.J. (2008). Soy isoflavone genistein in prevention and treatment of prostate cancer. Prostate Cancer Prostatic Dis..

[8] Deep G., Agarwal R. (2007). Chemopreventive efficacy of silymarin in skin and prostate cancer. Integr. Cancer Ther..

[9] Kren V., Walterova D. (2005). Silybin and silymarin-new effects and applications. Bimed. Papers..

[10] Taylor C.K., Levy R.M., Elliott J.C., Burnett B.P. (2009). The effect of genistein aglycone on cancer and cancer risk: A review of *in vitro*, preclinical, and clinical studies. Nutr. Rev..

[11] Wu Y., Fan Q., Lu N., Tao L., Gao Y., Qi Q., Guo Q. (2010). Breviscapine-induced apoptosis of human hepatocellular carcinoma cell line HepG2 was involved in its antitumor activity. Phytother. Res..

[12] Shon Y.H., Park S.D., Nam K.S. (2006). Effective chemopreventive activity of genistein against human breast cancer cells. J. Biochem. Mol. Biol..

[13] Delmonte P., Rader J.I. (2006). Analysis of isoflavones in foods and dietary supplements. J. AOAC. Int..

[14] Xiao C.W. (2008). Health effects of soy protein and isoflavones in humans. J. Nutr..

[15] Steiner C., Arnoulds S., Scalbert A., Manach C. (2008). Isoflavones and the prevention of breast and prostate cancer: New perspectives opened by nutrigenomics. Br. J. Nutr..

[16] Ouyang G., Yao L., Ruan K., Song G., Mao Y., Bao S. (2009). Genistein induces G2/M cell cycle arrest and apoptosis of human ovarian cancer cells *via* activation of DNA damage checkpoint pathways. Cell Biol. Int..

[17] Li Z., Li J., Mo B., Hu C., Liu H., Qi H., Wang X., Xu J. (2008). Genistein induces cell apoptosis In MDA-MB-231 breast cancer cells via the mitogen-activated protein kinase pathway. Toxicol. in Vitro.

[18] Moon Y.J., Shin B.S., An G., Morris M.E. (2008). Biochanin A inhibits breast cancer tumor growth in a murine xenograft model. Pharm. Res..

[19] Dave B., Eason R., Till S., Geng Y., Velarde C., Badger T., Simmen C. (2005). The soy isoflavone genistein promotes apoptosis in mammary epithelial cells by inducing the tumor suppressor PTEN. Carcinogenesis.

[20] Su S.J., Chow N.H., Kung M.L., Hung T.C., Chang K.L. (2003). Effects of soy isoflavones on apoptosis induction and G2-M arrest in human hepatoma cells involvement of caspase-3 activation, Bcl-2 and Bcl-XL downregulation, and Cdc2 kinase activity. Nutr. Cancer.

[21] Wang S., El-Deiry W.S. (2003). TRAIL and apoptosis induction by TNF-family death receptors. Oncogene.

[22] Holoch P.A., Griffith T.S. (2009). TNF-related apoptosis-inducing ligand (TRAIL): A new path to anti-cancer therapies. Eur. J. Pharmacol..

[23] Szliszka E., Czuba ZP., Domino M., Mazur B., Zydowicz G., Krol W. (2009). Ethanolic extract of propolis (EEP) enhances the apoptosis-inducing potential of TRAIL in cancer cells. Molecules.

[24] Szliszka E., Czuba Z.P., Jernas K., Krol W. (2008). Dietary flavonoids sensitize HeLa cells to tumor necrosis factor-related apoptosis-inducing ligand (TRAIL). Int. J. Mol. Sci..

[25] Horinanka M., Yoshida T., Shiraishi T., Nakata S., Wakada M., Nakanishi R., Nishino H., Sakai T. (2005). The combination of TRAIL and luteolin enhances apoptosis in human cervival cancer HeLa cells. Biochem. Biophys. Res. Comun..

[26] Szliszka E., Czuba ZP., Bronikowska J., Mertas A., Paradysz A., Krol W. (2009). Ethanolic extract of propolis (EEP) augments TRAIL-induced apoptotic death in prostate cancer cells. Evid. Based Complement. Alternat. Med..

[27] Szliszka E., Czuba Z.P., Mazur B., Sedek L., Paradysz A., Krol W. (2010). Chalcones enhance TRAIL-induced apoptosis in prostate cancer cells. Int. J. Mol. Sci..

[28] Jin C.Y., Park C., Cheong J., Choi B.T., Lee T.H., Lee J.D., Lee W.H., Kim G.Y., Ryu C.H., Choi Y.H. (2007). Genistein sensitizes TRAIL-resistant human gastric adenocarcinoma AGS cells through activation of caspase-3. Cancer Lett..

[29] Siegelin M.D., Gaiser T., Habel A., Siegelin Y. (2009). Daidzein overcomes TRAIL-resistance in malignant glioma cells by modulating the expression of the intrinsic apoptotic inhibitor, bcl-2. Neurosci. Lett..

[30] Banerjee S., Li Y., Wang Z., Sarkar F. (2008). Multi-target therapy of cancer by genistein. Cancer Lett..

[31] Dixon A., Ferreira D. (2002). Genistein. Phytochemistry.

[32] Kao T.H., Huang R.F.S., Chen B.H. (2007). Antiproliferation of hepatoma cell and progression of cell cycle as affected by isoflavone extracts from soybean cake. Int. J. Mol. Sci..

[33] Sakai T., Kogiso M. (2008). Soy isoflavones and immunity. J. Med. Invest..

[34] Johnson T.L., Lai M.B., Lai J.C., Bhushan A. (2008). Inhibition of cell proliferation and MAP kinase and akt pathways in oral squamous cell carcinoma by genistein and biochanin A. Evid. Based Complement. Alternat. Med..

[35] Liao S.Y., Chen J.C., Qian L., Shen Y., Zheng K.C. (2008). QSAR action mechanism and molecular design of flavone and isoflavone derivatives with cytotoxicity against HeLa. Eur. J. Med. Chem..

[36] Feng L., Awale S., Tezuka Y., Kadota S. (2008). Cytotoxic constituents-from Brazilian red propolis and their structure-activity relationship. Bioorg. Med. Chem..

[37] Khaosaad T., Krenn L., Medjakovic S., Rannen A., Lössl A., Nell M., Jungbauer A., Vierheilig H. (2008). Effect of mycorrhization on the isoflavone content and the phytoestrogen activity of red clover. J. Plant Physiol..

[38] Zhou N., Yan Y., Li W., Wang Y., Zheng L., Han S., Yan Y., Li Y. (2009). Genistein inhibition of topoisomerase IIα expression Participated by Sp1 and Sp3 in HeLa Cell. Int. J. Mol. Sci..

[39] Blay M., Espinel A.E., Delgado M.A., Baiges I., Blade C., Arola L., Salvado J. (2010). Isoflavone effect on gene expression profile and biomarkers of inflammation. J. Pharm. Biomed. Anal..

[40] Veitch N.C. (2007). Isoflavonoids of the leguminosae. Nat. Prod. Rep..

[41] Zhao L.H., Huang C.Y., Shan Z., Xiang B.G., Mei L.H. (2005). Fingerprint analysis of *Psoralea corylifolia* by HLPC and LC-MS. J. Chromatogr..

[42] Latha P.G., Evans D.A., Panikkar K.R., Jayavardhanan K.K. (2000). Immunomodulatory and antitumour properties of *Psoralea corylifolia* seeds. Fitoterapia.

[43] Kim S.H., Kim S.H., Lee S.C., Song Y.S. (2009). Involvement of both extrinsic and intrinsic apoptotic pathways in apoptosis induced by genistein in human cervical cancer cells. Ann. N.Y. Acad. Sci..

[44] Lee M.W., Bach J.H., Lee D.Y., Joo W.S., Kim K.Y., Lee W.B., Kim S.S. (2005). The activation of ERK1/2 *via* a tyrosine kinase pathway attenuates trail-induced apoptosis in HeLa cells. Cancer Invest..

[45] Siegelin M.D., Siegelin Y., Habel A., Gaiser T. (2009). Genistein enhances proteosomal degradation of the short isoform of FLIP in malignant glioma cells and thereby augments TRAIL-mediated apoptosis. Neurosci. Lett..

[46] Nozawa F., Itami A., Saruc M., Kim M., Standop J., Picha K.S., Cowan K.H., Pour P.M. (2004). The combination of tumor necrosis factor-related apoptosis-inducing ligand (TRAIL/Apo2L) and genistein is effective in inhibiting pancreatic cancer growth. Pancreas.

[47] Jin C.Y., Park C., Lee S.J., Kim W.J., Choi Y.H. (2009). Genistein enhances TRAIL-induced apoptosis through inhibition of p38 MAPK signaling in human hepatocellular carcinoma Hep3B cells. Chem. Biol. Interact..

[48] Jin C.Y., Park C., Moon S.K., Kim G.Y., Kwon T.K., Lee S.J., Kim W.J., Choi Y.H. (2009). Genistein sensitizes human hepatocellular carcinoma cells to TRAIL-mediated apoptosis by enhancing Bid cleavage. Anticancer Drugs.

[49] Suetsugi M., Su L., Karlsberg K., Yuan YC., Chen S. (2003). Flavone and isoflavone phytoestrogens are agonists of estrogen-related receptors. Mol. Cancer Res..

[50] Szliszka E., Bronikowska J., Majcher A., Miszkiewicz J., Krol W. (2009). Enhanced sensitivity of hormone-refractory prostate cancer cells to tumor necrosis factor-related apoptosis-inducing ligand (TRAIL) mediated cytotoxicity by taxanes. CEJ Urol..

